# Reply to: Craniofacial morphology does not support a pre-contact Carib “invasion” of the northern Caribbean

**DOI:** 10.1038/s41598-021-95560-z

**Published:** 2021-08-20

**Authors:** Ann H. Ross, William F. Keegan, Michael P. Pateman, Colleen B. Young

**Affiliations:** 1 Department of Biological Sciences, North Carolina State University, Raleigh, NC 27695 USA; 2 Caribbean Archaeology, Florida Museum of Natural History, University of Florida, Gainesville, FL 32611 USA; 3 AEX Bahamas Maritime Museum, Freeport, Grand Bahama Bahamas; 4 Department of Anthropology, University of Missouri, Columbia, MO 65203 USA

**Keywords:** Biological anthropology, Archaeology

**replying to**: C. M. Giovas et al.; *Scientific Reports*https://doi.org/10.1038/s41598-021-95558-7 (2021).

## Introduction

We are pleased that our research has generated a much-needed dialogue on the prehistory of the Caribbean and appreciate the opportunity to address the misunderstandings reflected in comments by Giovas and colleagues^1^. Five issues are addressed: (1) misuse of non-metric multidimensional scaling analysis (NMMDS); (2) their erroneous representation of our use of ethnohistoric accounts and archaeological data; (3) their conflation of biology and “ethnicity”; (4) sample size; (5) chronology; and we begin by reiterating the project goal.

Our initial objective was to determine whether the pre-Columbian communities of The Bahamas originated in Hispaniola or Cuba using facial morphology (Keegan and Hofman^2^), based on previously observed significant differences between those islands (Ross and Ubelaker^3^). The clusters were derived using a Mahalanobis or generalized distance matrix, a function of the group means and the pooled variances and covariances (Afifi and Clark^4^), computed using the Principal Components Analysis (PCA), a dimension reducing technique, derived from the craniofacial anatomical landmarks (e.g., coordinate data).

**Figure 1:**
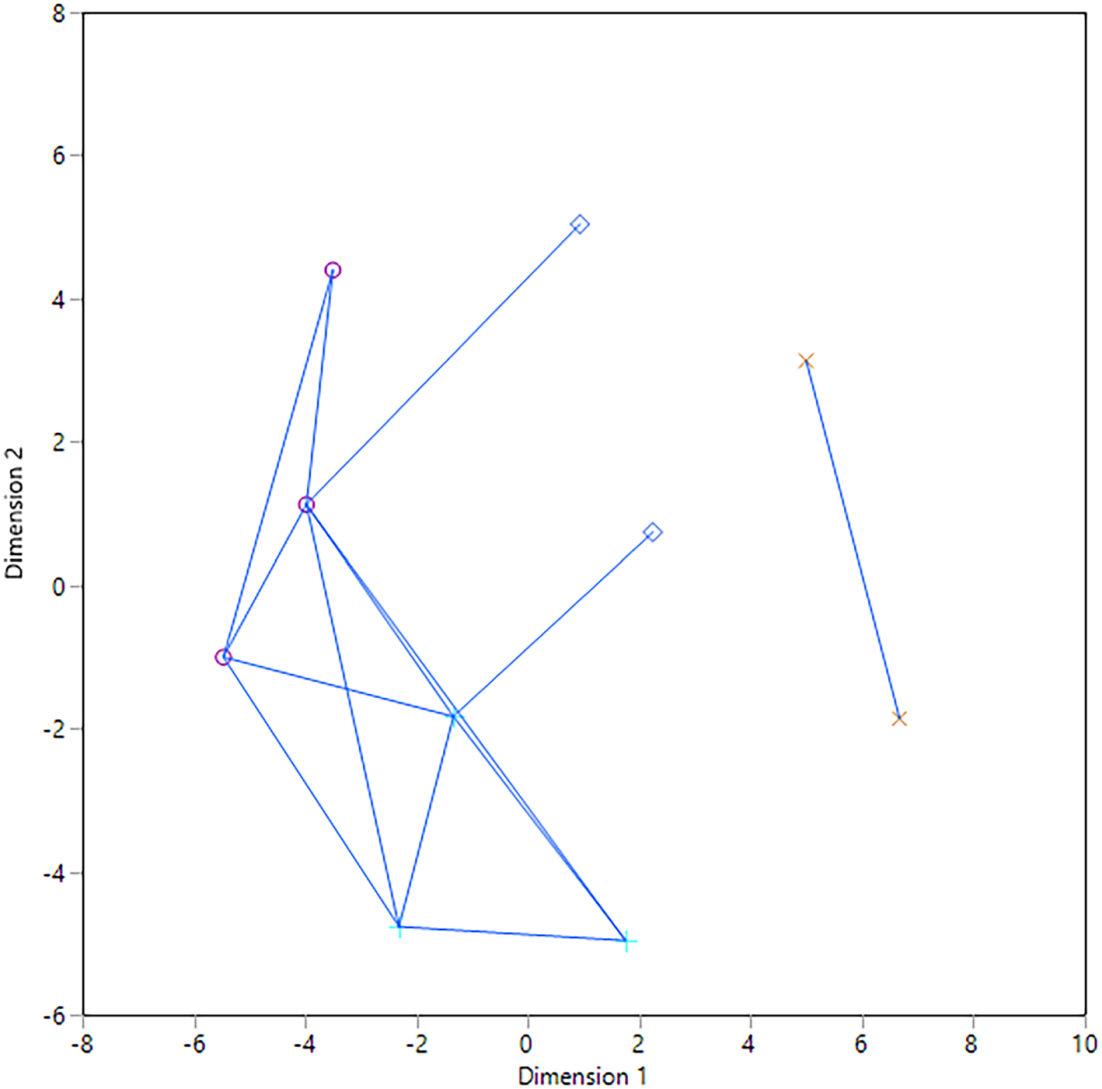
MDS visual check using Waern of actual and predicted proximities shows it is not a good representation.

**Figure 2:**
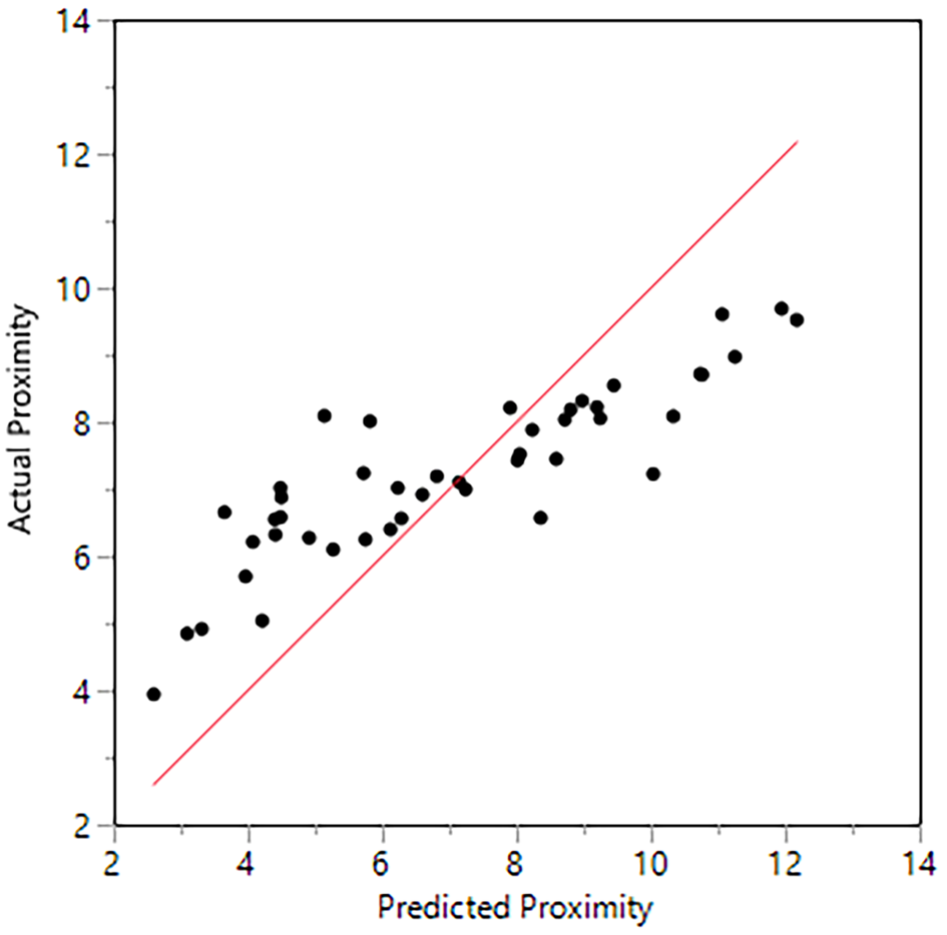
Shepard diagram, a plot of actual and predicted proximities, shows that it is not a good representation of group similarities.

1. We reiterate the importance of the tests we used for craniofacial analyses. We analyzed facial shape and size using coordinate data and geometric morphometrics (GMM) including male and female individuals based on valid statistical methods. We used accepted methods to study population history, movements, and biological distances (agglomerative hierarchical cluster, spatial analyses, etc.; see Sneath and Sokal^5^). The approach we contribute is the application of a population structure perspective (Sneath and Sokal^5^) coupled with an archaeological and historical context—providing a more holistic lens.
  In response, Giovas et al. offer a figure of non-metric multidimensional scaling analysis (NMMDS), an ordination method, which is simply a means to visualize and graphically illustrate the Mahalanobis distance matrix published in our study. With this plot, they contest the results of our in-depth population study. NMMDS is an exploratory analysis often used in conjunction with clustering methods to quickly visualize the distance matrix (JMP multivariate methods, Neves et al.^6^, Stynder and Ackermann^7^; Lessa^8^; von Cramon-Taubadel et al.^9^). Their figure does not equate to the hierarchical average linkage cluster analysis we conducted (see original paper for details). In addition, they misinterpret our generalized distance (a classic measure of biological distance, Pietrusewsky^10^) by using NMMDS, which is a dimension reducing technique (e.g., concerned with finding a low dimension from high dimensional data such as coordinate data, Härdle and Simar^11^). Principal component analysis or PCA is the preferred dimension reducing technique in GMM that was applied in our original study. Generalized (Mahalanobis) distance is used to test whether group centroids are significantly different (Ross^12–14^) and is used to examine the patterning of phenetic (morphometric) affinities and not to reduce the dimensionality of the data.
  Notably, MDS can obscure the underlying phenetic affinities, especially when assessing interspecific relationships and they may not disclose finer divisions that are biologically significant (Sneath and Sokal^5^, Neves et al.^6^, Lessa^8^). To illustrate this, we reproduced the non-metric multidimensional scaling analysis in JMP 14.3 using the Waern links as a visual check of the actual and predicted proximities (JMP multivariate methods, Waern^15^, Fig. 1). Figure 1 shows that non-metric MDS is not a good representation of the proximities based on the links between the Cuban and Yucatan samples because they stretch across the plot to the other samples and by the unconnected link for Florida and Panama to the rest of the groups. We further tested this using a Shepard diagram (a plot of actual and predicted proximities, Fig. 2) that shows that it is not a good representation of group similarities as the underlying assumption NMMDS is that there is a monotonic relationship (i.e., increase in distance from the most to the least similar pair) between the actual and predicted values and the Shepard diagram is non-monotonic. A good representation would be indicated by the points falling on the x = y line (red line). The reported r^2^ value of 0.76 for the non-metric MDS further demonstrates that this is not a good fit to explain the amount of variation within the data (Fig. 2). These procedural statistical tests demonstrate that their MDS plot does not accurately represent our data and that the assumptions used in their NMMDS test to produce their results and interpret their findings (such as the number of dimensions or starting location) may be erroneous. Further, in our original manuscript, we established that there was a lack of spatial autocorrelation for shape and a non-monotonic pattern for centroid size supporting the inappropriateness of using NMMDS for our data (see Figure 7 in our original paper).
  In conclusion, the non-metric MDS plot presented by Giovas et al.^1^ does not accurately capture the clusters created by our thorough population structure approach that included multivariate, cluster, and spatial analyses, and does not pass the non-metric MDS visual Waern and Shepard proximity checks. Thus, their statistical critique is based on inappropriate methods and procedures, and in our view should be rejected.
2. The morphological data provide a new way to conceptualize the Indigenous communities of the northern Caribbean. It was only after the craniofacial clusters were generated that we recognized the common material culture shared by the Hispaniola/Jamaica/Bahamas (HJB) cluster was Meillacoid pottery.
  The HJB cluster encompasses the region in which Meillacoid series pottery predominates (Keegan and Hofman^2^). Rouse^16^ derived Meillacoid style pottery from an Ostionoid tradition based largely on the red paste observed near Ft. Liberté, Haiti, where he conducted his primary fieldwork (Rouse^17^). He further concluded that the incised designs reflected the copying of motifs on Archaic Age stone bowls. We are not convinced that Meillacoid developed from an Ostionoid tradition. Meillacoid pottery appeared suddenly in Hispaniola as a full-blown complex of motifs created through a combination of incision, punctation, appliqué, and modeling. These motifs are far more complicated than could be achieved by simply copying Archaic designs, and they are fully represented in pottery series from western South America, including “Carib” (Lathrap^18^) and Valdivia (Meggar et al.^19^).
  Outside Ft. Liberté, the paste has a much darker color (fired in a reducing as opposed to the oxidizing environment), and there is little evidence for a transition from Ostionoid to Meillacoid, especially in Jamaica (Keegan^20^). There also is no evidence for a transition from Meillacoid to Chicoid, as Rouse proposed; the two were contemporaneous and independent. We now know that Meillacoid ceramics continued in use until Contact, and Meillacoid motifs have been observed at a late period site in Puerto Rico (Morsink, personal communication^5,21^) and on St. John, USVI (Wild^22^). Although none of the respondents conduct research in the Meillacoid area, they reject, without evidence, the empirically grounded conclusions of Dominican and Venezuelan archaeologists (Zucchi^23,24^; Veloz Maggiolo et al.^25^). Giovas et al.^1^ mention specifically that these conclusions were first proposed over 30 years ago, but the conventional understanding of Meillacoid is based on research conducted 80 years ago. Recent research with novel techniques is transforming our understanding of the communities that made and used Meillacoid pottery (Keegan and Hofman^2^).
  At issue is why a coherent ceramic assemblage that shares an abundance of modes with western South America suddenly appeared in Hispaniola. We proposed a migration from western Venezuela, whose timing coincides with the expansion of Caribs in that area, which is based on phenotypic characteristics that clustered in HJB. This migration could have involved a large-scale population expansion or the infiltration of smaller numbers of men (Lathrap^18^; Schmidt^26^). We chose to call them Carib based on Spanish accounts of “Caribs” in the Greater Antilles and the Bahamas. Guinea pigs were mentioned only to highlight their geographical origins and the timing of their introduction coincident with our proposed migration; any further inference is limited by the small sample size.
3. Giovas and colleagues^1^ conflate biology and culture (see Crellin and Harris^27^), and compound their error by claiming filiation between present-day Kalinago and Garifuna and the Meillacoid “Caribs” of HJB. Garifuna is an Afro-indigenous group that was not present before the Trans-Atlantic Slave Trade. We made no claims concerning language or “ethnicity.” The name “Carib” has been used in so many ways that it seems to create endless confusion. Columbus mentioned “Caribs” while sailing through the Bahamas and along the north coasts of Cuba and Hispaniola during his first voyage (Dunn and Kelley^28^). Other Spanish chroniclers continued to do likewise (Keegan^29^). Giovas et al. acknowledge the substantial differences between the cultures of the Greater Antilles and those of the southern Lesser Antilles, yet they insist that Columbus must have been describing the inhabitants of the southern Lesser Antilles, who were living over 1500 km away. He wasn’t^2^. We included a discussion of previous accounts by early chroniclers to draw attention to the long-neglected subject of Caribs in the Greater Antilles. Further, they continue to argue that we used our analysis to support “ethnic” distinctiveness and reiterate that this is a misrepresentation of the original work. They refer to the individuals used in our study as “specimens”, which shows a lack of sensitivity for the decedents. This is a community collaborative project whereby archaeological research and excavations have been approved and provided by present-day community members in the Bahamas to elucidate the prehistory of the Caribbean. They also claim that we are harming contemporary populations of the Lesser Antilles whilst we only examined prehistoric biological variation in the Western Antilles with no living descendants. If anything, associating the Island Carib with the pejorative attitudes of the Spanish, who were writing about completely different Indigenous communities may be more harmful.
4. We agree that sample size is an issue for island archaeologists, particularly for those who work in coastal environments, such as the Caribbean (Erlandson and Fitzpatrick^30^; Ross and Cunningham^31^), which is why such few studies have been reported from this region before but contend that the samples we have and use are a valid depiction of biological (i.e., genetic) similarity. Sample size was limited by the number of human crania from The Bahamas with complete facial features. While there is always a need for more individuals, any science that studies the past is limited by the finite material left by time. Caribbean archaeology, in particular, is wrought with problems of preservation due to the loss of sites via sea-level rise, development, tourism, sand mining, and erosion (Erlandson and Fitzpatrick^30^); and rapid degradation of skeletal material in a tropical environment (Ross and Cunningham^31^). The samples we have are remarkable in their preservation, despite these variables, and allow us to extract necessary craniofacial measurements to elucidate critical information on the people of this region. Craniofacial analyses do not necessitate large sample sizes to extract meaningful biological information and by the dimension reducing technique, PCA that was applied before subsequent multivariate statistical analyses (Neves et al.^6^; González-José et al.^32^; Powell and Neves^33^). We plan to digitize additional individuals as these become available.
5. Chronology via precise dating methods is important for time-sensitive conclusions, such as date of migration, or environmental and cultural sequencing. We are assessing the biological relatedness among individuals through space prior to European colonization. The samples used are of pre-Columbian individuals from 0 to 1500 AD. Giovas et al. focus on potentially non-local Chichén Itzá samples “possibly originating from as far away as Central America and Mexico’s Central Highlands” as a critique for our skeletal samples. While we understand the point they are trying to make, and agree that further dating and isotopic studies will elucidate the unique individual histories of skeletal samples, the individuals we sampled accurately represent the spatial and temporal scale we are studying. Again, our study aimed to understand Cuba and Hispaniola biological contributions to pre-colonial Bahamians. The detected Carib contributions were accurately assessed with appropriate comparative samples from South America. Additionally, a total of 121 new AMS dates from human remains are reported in recent genetic studies, along with an additional 65 AMS dates for The Bahamas (Hanna et al., forthcoming^34^; Sullivan et al.^35^; Schulting et al.^36^). Our study includes New World individuals after initial settlement, within relevant geographic areas, and thus, our time and spatial frame to explore the underlying population structure are appropriate (Ross and Ubelaker^11^).

In conclusion, we collected for the first time 3D measurements of Lucayan facial morphology and compared these individuals to established datasets from the circum-Caribbean. Using standard methods, we observed clustering that identified a significant relationship among individuals living in Hispaniola, Jamaica, and The Bahamas. The material culture shared by these individuals is represented by Meillacoid pottery. The results highlight a connection between western Venezuela and the Antilles that demands further investigation.
